# Using RNA sequencing to characterize female reproductive genes between Z and E Strains of European Corn Borer moth (*Ostrinia nubilalis*)

**DOI:** 10.1186/1471-2164-15-189

**Published:** 2014-03-12

**Authors:** Nooria Al-Wathiqui, Sara M Lewis, Erik B Dopman

**Affiliations:** Department of Biology, Tufts University, Medford, MA 02155 USA

**Keywords:** Female reproductive genes, Speciation, Next-generation sequencing, Lepidoptera

## Abstract

**Background:**

Reproductive proteins often evolve rapidly and are thought to be subject to strong sexual selection, and thus may play a key role in reproductive isolation and species divergence. However, our knowledge of reproductive proteins has been largely limited to males and model organisms with sequenced genomes. With advances in sequencing technology, Lepidoptera are emerging models for studies of sexual selection and speciation. By profiling the transcriptomes of the bursa copulatrix and bursal gland from females of two incipient species of moth, we characterize reproductive genes expressed in the primary reproductive tissues of female Lepidoptera and identify candidate genes contributing to a one-way gametic incompatibility between Z and E strains of the European corn borer (Ostrinia nubilalis).

**Results:**

Using RNA sequencing we identified transcripts from ~37,000 and ~36,000 loci that were expressed in the bursa copulatrix or the bursal gland respectively. Of bursa copulatrix genes, 8% were significantly differentially expressed compared to the female thorax, and those that were up-regulated or specific to the bursa copulatrix showed functional biases toward muscle activity and/or organization. In the bursal gland, 9% of genes were differentially expressed compared to the thorax, with many showing reproduction or gamete production functions. Of up-regulated bursal gland genes, 46% contained a transmembrane region and 16% possessed secretion signal peptides. Divergently expressed genes in the bursa copulatrix were exclusively biased toward protease-like functions and 51 proteases or protease inhibitors were divergently expressed overall.

**Conclusions:**

This is the first comprehensive characterization of female reproductive genes in any lepidopteran system. The transcriptome of the bursa copulatrix supports its role as a muscular sac that is the primary site for disruption of the male ejaculate. We find that the bursal gland acts as a reproductive secretory body that might also interact with male ejaculate. In addition, differential expression of proteases between strains supports a potential role for these tissues in contributing to reproductive isolation. Our study provides new insight into how male ejaculate is processed by female Lepidoptera, and paves the way for future work on interactions between post-mating sexual selection and speciation.

**Electronic supplementary material:**

The online version of this article (doi:10.1186/1471-2164-15-189) contains supplementary material, which is available to authorized users.

## Background

Sexual selection is a powerful evolutionary force that can drive species divergence [1–4]. Although many studies focus on how organisms choose mates during pre-mating sexual selection, the process is not limited to courtship, but rather occurs across multiple time points before, during, and after copulation [5–7]. Post-mating sexual selection has proven challenging to study because it can involve interactions between the female reproductive tract and the male ejaculate on a molecular level. Such interactions include male-male sperm competition, sexual conflict involving male and female proteins, and cryptic female choice. Sexual conflict is of particular interest because it arises from the divergent reproductive interests of males and females and thus may represent an important component of post-mating interactions [8]. Many mechanisms of post-mating sexual selection involve co-evolutionary arms races between the sexes, a process that can lead to rapid trait evolution within [8, 9] or divergence between populations [10–12]. Differences in reproductive traits between populations can quickly result in post-mating, pre-zygotic barriers [13], potentially playing a powerful role in species formation.

Most work on post-mating, pre-zygotic barriers has focused on interactions between the female reproductive tract and male sperm. Specifically, cryptic female choice has been identified as a possible mechanism for conspecific sperm precedence, in which multiply mated females produce more offspring sired by conspecific rather than heterospecific mates [14–16]. Conspecific sperm precedence is widespread and has been demonstrated in many insect species, including fruit flies, ground crickets and flour beetles [14, 16–19]. Of several possible mechanisms underlying conspecific sperm precedence in *Drosophila mauritiana* and *Drosophila simulans*, biased sperm use by females was found to be a key determinant [20]. Female *Drosophila* are able to favor conspecific males by preferentially storing sperm in separate storage organs [20]. Although multiply mated females seem to be able to bias paternity of their offspring, the nature of the interactions between male and female reproductive proteins that might lead to such differential sperm use is unclear [21, 22].

After mating, the female reproductive tract interacts not only with male sperm, but also with seminal fluid. In many taxa, male reproductive proteins are produced in accessory glands or the ejaculatory duct, which are then transferred to the female as components of the male ejaculate. Collectively, these non-sperm components of the ejaculate are called seminal fluid proteins and they are quite numerous [23]. In fruit flies, *Drosophila melanogaster* males produce over 100 seminal proteins that are transferred to females during mating [24–26]. These proteins have profound effects on female behavior and physiology, including changes in lifespan, ovulation, feeding habits and sperm storage patterns [14, 24, 26]. Not only do male reproductive proteins have important effects on females, they are potentially powerful drivers of post-mating, pre-zygotic reproductive isolation because many of them evolve rapidly [22]. Seminal fluid proteins have been comprehensively characterized in several insect taxa, including fruit flies, mosquitoes, honeybees, crickets, flour beetles, butterflies, and bedbugs [24, 27–35]. In contrast, we know very little about the many possible interacting female reproductive proteins for any one species.

Although the reproductive tracts of female insects also contain secretory tissue [36], to date female reproductive genes have been comprehensively studied in very few taxa including mosquitos, fruit flies, and honeybees [37–44]. Unsurprisingly, female reproductive genes have been best characterized in *Drosophila* species including: *D. melanogaster*, *D. simulans, D. arizonae* and *D. mojavensis*[38–43]. Many of these investigations have identified proteases and protease inhibitors, as well as genes related to muscle activity, immune response, and energy metabolism in female reproductive tracts [38–43]. Genes with these functions are predicted to mediate interactions with male ejaculate after mating. Indeed, muscle activity is a key component of female-mediated sperm storage and ejaculate processing [45, 46], while proteases and protease inhibitors have been shown to be required for activation of ovulation-inducing seminal fluid proteins in *D. melanogaster*[47]. Furthermore, immune and energy metabolism genes appear to be important for the demands of egg production and oviposition or to protect females from male-introduced pathogens [38, 40, 48]. Many relevant female reproductive proteins are secreted from female tissue or are transmembrane, as these are likely to directly interact with male ejaculate or act as receptors for male seminal fluid proteins [42].

To reach a comprehensive understanding of the mechanisms by which male and female reproductive genes drive post-mating sexual selection and ultimately species divergence, studies of reproductive proteins must consider organisms with different mating systems and different levels of sexual conflict. As the second largest insect order with ~170,000 known species, moths and butterflies comprising the order Lepidoptera are ideal for study because the degree of multiple mating by females, and thus the opportunity for sexual conflict, positively correlates with speciation rate [49, 50]. However, very little is known about male and female reproductive genes in Lepidoptera. Two comprehensive studies, both in Heliconid butterflies, have identified male reproductive proteins in lepidopterans [34, 35]; however, researchers have yet to identify female genes from any structure in the lepidopteran female reproductive tract.

**Figure 1:**
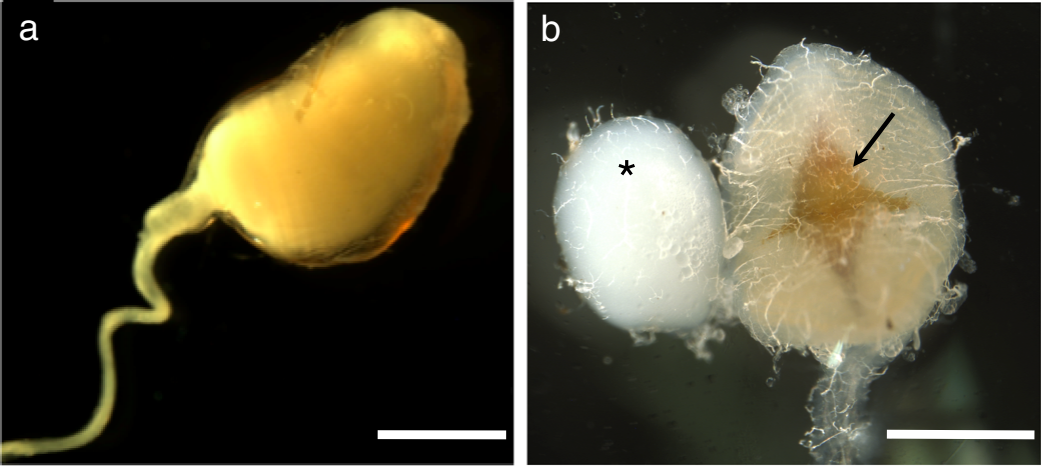
**Male and female ECB reproductive structures. a**. An ECB male spermatophore transferred to a female upon mating. **b**. The virgin female bursa copulatrix with signum (indicated by arrow) and the bursal gland (indicated by*). Notice that even in virgins the bursal gland is filled with fluid. Scale bars represent 1 mm.

Most male lepidopterans transfer their ejaculate in a package called a spermatophore (Figure 1a) [36, 49]. Although produced by males, the spermatophore is actually formed inside a large, sac-like structure inside the female called the bursa copulatrix (Figure 1b). Inside the bursa copulatrix, the spermatophore is broken open by the signum, a chitinized structure embedded in the muscular wall of the bursa copulatrix, and both sperm and male seminal fluid proteins are released into the female reproductive tract; however, the spermatophore remains in the bursa copulatrix as a visible structure for the entirety of the females life [36, 49]. Thus, the bursa copulatrix represents a strong candidate arena for the resolution of sexual conflict and the origin of post-mating, pre-zygotic isolation. In general, males that prevent females from remating will achieve greater paternity success. In species where this is true, male traits will evolve that delay female remating. For example, males that transfer larger spermatophores are able to delay female remating for longer in some lepidopterans [51, 52] and in *D. melanogaster*, identified male reproductive proteins act to reduce female receptivity to future mates [47, 53]. On the other hand, females in many taxa gain material and genetic benefits from multiply mating [54, 55], and therefore selection will favor morphological and biochemical traits that allow females to rapidly process male spermatophores. Although recent microstructural studies in Lepidoptera suggest the bursa copulatrix could have a secretory function, studies have yet to characterize any secretions from the structure [56]. Such secretions could be important for breaking down spermatophores or for interacting with male reproductive proteins.

A second structure found in some lepidopteran females that could mediate within- and between-species mating success is the bursal gland. Although patterns of evolutionary conservation remain unclear, the bursal gland is a prominent anatomical feature of the female reproductive system in the European corn borer moth, *Ostrinia nubilalis*[57]. The bursal gland is dorsally connected directly to the bursa copulatrix by a short duct (Figure 1b) and is approximately 0.5-0.8 mm in diameter. In virgin females the bursal gland is filled with a white, translucent fluid, which then flows into the bursa copulatrix under pressure [57]. After mating, the bursal gland is similarly filled, but with an opaque fluid [57]. The function of this gland is currently unknown, however its proximity and direct connection to the bursa copulatrix and male spermatophore suggests that the bursal gland could function during spermatophore breakdown or to secrete female reproductive proteins regulating the activity of male reproductive proteins.

Here, we use next-generation RNA sequencing to characterize gene expression in the female bursa copulatrix and bursal gland as the first step towards determining how these tissues are involved in post-mating, pre-zygotic isolation in the European corn borer moth (hereafter, “ECB”). The Z and E strains of ECB are emerging textbook models for the study of speciation [13], in which the two incipient species split approximately 75,000 to 150,000 years ago through the evolution of manifold reproductive barriers [58, 59]. Females of both strains mate multiply [57] and suffer reduced longevity after mating [60], conditions that are generally favorable for sexual conflict and the evolution of post-mating, pre-zygotic isolation. Consistent with this notion, one of seven barriers between strains, accounting for a ~30% reduction in gene flow, stems from reduced lifetime fecundity following between-strain mating. This post-mating, pre-zygotic incompatibility is asymmetric: Z-strain females that have mated with E-strain males lay significantly fewer eggs over their lifetime [59]. However, the mechanism underlying this gametic isolation is unknown [59]. By examining the transcriptome of bursa copulatrix and bursal gland reproductive tissues within and between ECB strains, we characterize candidate genes that could be contributing to the egg-laying dysfunction. Specifically, we characterize the function of the bursa copulatrix and bursal gland and identify female reproductive genes that may be involved in isolation using the following criteria: (1) putative proteins that are secreted or membrane bound, (2) an up-regulation of transcripts that aid in muscle contraction and that may assist in spermatophore breakdown or sperm transfer to storage, (3) an up-regulation of proteases and protease inhibitors that could mediate male seminal fluid protein potential, and (4) differential expression between Z and E strains.

## Methods

### Sample preparation and sequencing

We collected bursa copulatrix and bursal gland tissues from 2-day old adult Z- and E-strain ECB females (n = 12 per strain, Figure 1). At this stage females are reproductively mature [60, 61]. As the goal of this study was to identify the reproductive function of these two structures and characterize differences between ECB strains and not to identify genes directly affected by mating, all female tissues were collected from virgins. The following dissections were done in RNAlater (Qiagen, California). First, females were sacrificed and the bursa copulatrix and attached bursal gland were removed from an incision in the female abdomen. Next, fat body was removed from both structures and, after separating the bursal gland from the bursa copulatrix, both tissues were stored in RNAlater at -80°C. After tissue collection, total RNA was extracted from bursa copulatrix and bursal gland tissues using an RNeasy Midi kit (Qiagen, California). Bursal glands and bursa copulatrix tissues from four females were pooled by strain into three separate samples for each tissue type and each strain prior to an initial tissue homogenization step. This resulted in twelve samples, three bursal gland samples and three bursa copulatrix samples for each strain. RNA quantities were assessed using a Nanodrop and 1 μg of total RNA from each sample was used to create cDNA libraries (Illumina Truseq RNA sample preparation kit v2, San Diego, CA). To prepare samples for sequencing, mRNA was selected from each sample using poly-T-tail magnetic beads. Next, cDNA was synthesized using Superscript II (Invitrogen, Grand Island, NY) and Illumina adapters were attached to libraries for multiplexing prior to sequencing. cDNA strands were then amplified using 15 PCR cycles. Next, quality and quantity of cDNA was confirmed using a NanoDrop (Thermo Fisher Scientific Inc., Delaware) and an Agilent 2100 Bioanalyzer (Santa Clara, CA). Due to low sample quality for one Z-strain bursa copulatrix library, we did not sequence this sample, which left us with two Z-strain bursa copulatrix tissue libraries. Four Z- and E- bursal gland and bursa copulatrix cDNA libraries were multiplexed and sequenced in each of three lanes of two Illumina flow cells. Libraries were sequenced using an Illumina HiSeq2000 with 50 bp single-end reads. To evaluate tissue-biased expression, we took advantage of previously sequenced Z- and E- female thorax libraries developed during a separate project. Z-strain and E-strain female thoraxes were collected from 2 females each. Briefly, female thorax cDNA libraries were created using the SMART cDNA library 6.7 protocol (Takara Bio, Otsu, Shiga, Japan). These libraries were sequenced using an Illumina GA IIx and 40×40 bp paired-end reads with a 200 bp insert length.

### Data preprocessing

After sequencing, all reads were subjected to quality control and trimming using Trimmomatic v0.17 to remove Illumina sequencing adapters and low quality reads [62]. Leading and trailing bases with a quality score < 5 were trimmed from reads and then each read was trimmed by a sliding window with a width of 4 bp and minimum average quality of 15. After adapter and quality trimming, only reads ≥ 36 bp were retained.

Although we used magnetic beads to select for mRNA, our samples still contained small amounts of mtDNA and rRNA sequences. To remove these contaminants we used the short read aligner Bowtie 2 [63]. Bowtie2 uses the Burrow-Wheeler transformation to index a reference, then searches the index until it finds an alignment for a specific read [63]. We aligned our RNAseq reads to the complete ECB mitochondrial genome (NC_003367.1), and all published ECB ribosomal sequences [AF336303.1, AF077013.1, DQ988989.1, AB568463.1, AY513653.1, JX683305.1, JX683313.1, AB568278.1, AB568276.1, AB568274.1, EU532443.1, EU532441.1, EU532439.1, EU532444.1, EU532442.1, EU532440.1, EU532438.1, AF349036.1] in the NCBI database using default parameters and removed these sequences. Identical reads were then collapsed using FastX toolkit to reduced library complexity and decrease the computational needs for transcriptome assembly [64].

### De novo sequence assembly

We used the Trinity program suite to assemble all 13 tissue libraries including female bursa copulatrix and bursal gland libraries, as well as the two thorax libraries into a single assembly [65]. Trinity uses the inchworm, chrysalis, and butterfly software modules to create a de novo assembly. First, inchworm assembles reads into unique sequences. Chrysalis then clusters sequences into contiguous sequences and a de Bruijn graph is created for each cluster of contiguous sequences. Lastly, butterfly uses the de Bruijn graphs to construct transcripts. For all steps, we used settings recommended by developers, including merging the assembly at a kmer of 25 [65, 66]. Finally, following de novo assembly, we selected the longest transcript at each locus to eliminate redundancy.

### Annotation

To annotate our assembled transcriptome, we used the program Blast2Go [67, 68]. First, putative homologs were identified by performing a blastx search of the entire NCBI non-redundant protein database (e-value cutoff 10^-3^). For all sequences with significant blast hits, four different mappings were conducted. First, BLAST accession numbers are used to find gene names and symbols from NCBI gene_info and gene2accession. Then, gene_info identifiers are used to retrieve UniProt IDs using PSD, UniProt, SwissProt, TrEMBL, RefSeq, GenPept and PDB databases. In the final two steps, BLAST accessions were searched in the dbxreftable and the gene product table of the GO database. Finally, Blast2go computed an annotation score for all possible GO terms for each sequence [67, 68].

### Differential expression analysis

To identify differentially expressed sequences, we first mapped our reads back to our assembled transcriptome using Bowtie 2. For read mapping, we used the 'very sensitive’ setting in Bowtie 2 because preliminary trials indicated that this setting resulted in the most uniquely aligned reads. Differences between the SMART and TRUseq library preparation protocols could potentially lead to biases related to GC content, read length, and sequencing depth in each library. To help control for these and other possible biases, we then normalized our libraries prior to differential expression analysis using the programs EDAseq [69]. EDAseq performs within-lane normalization to account for differences in gene length and GC content and between-lane normalization to account for differences in sequencing depth [69]. Within-lane normalization uses global scaling normalization, which separates genes into equally sized bins based on GC-content and then matches different parameters of the count distribution across bins. For between- lane normalization, EDAseq uses a full-quantile normalization procedure that forces equal library sizes across lane.

**Figure 2:**
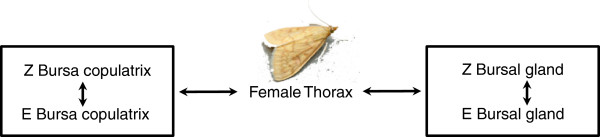
**Differential expression analysis tissue comparisons.** Our generalized linear model used to calculate differentially expressed genes between E- and Z- strain ECB included the following comparisons: female thorax versus the female bursa copulatrix, female thorax versus bursal gland, Z-strain versus E-strain bursa copulatrix, and Z-strain versus E-strain bursal glands.

After normalized read counts were obtained, we used the R package, edgeR to identify differentially expressed genes for all comparisons of interest using the normalized counts for each library (Figure 2) [70–73]. edgeR uses empirical Bayes methods to estimate gene-specific variation. As we were interested in four comparisons in particular, we used a generalized linear model approach in which we assessed differential expression with strain and tissue type as factors (Figure 2). Our model did not include an interaction term. Finally, a GLM likelihood ratio-test was used to identify differentially expressed genes [70]. Genes were considered differentially expressed if they had a false-discovery rate (FDR) of <0.01.

### Characterizing bursa copulatrix and bursal gland function

We used a three-pronged approach to characterize the reproductive function of the bursa copulatrix and the bursal gland. First, we adopted a common method to determine the specific functions of these tissues by ignoring housekeeping genes that have similar expression profiles across reproductive and non-reproductive tissues. For all of the remaining transcripts with significant expression differences between the bursa copulatrix and thorax, or between the bursal gland and thorax, gene annotations were pulled and enriched/depleted gene ontology categories were identified using a two-tailed fisher’s exact test in Blast2go with a term filter cutoff of FDR ≤ 0.05. Our entire non-redundant transcriptome containing transcripts from all three tissue types was used as the null distribution of GO categories.

Second, we identified signal peptides and transmembrane helices from the bursa copulatrix and bursal gland non-redundant transcriptome. For the purpose of identifying secreted and transmembrane proteins in the bursa copulatrix and bursal gland, we used a tblastx to remove all thorax sequences from our transcriptome. Next, to estimate predicted protein sequence from female bursa and bursal gland RNA-seq libraries, we used ESTscan [74], in which biases in hexanucleotide usage in coding versus non-coding regions and a Hidden Markov Model are used to predict protein-coding sequences. Subsequently, we identified sequences containing a secretion motif using SignalP 4.0 [75], which uses a neural network-based method to identify signal peptides. We then used TMHMM 2.0 to identify sequences with transmembrane helices [76], in which a Hidden Markov Model is used to predict integral membrane proteins.

Our last step to characterize the function of the bursa copulatrix and the bursal gland was to identify putative ECB homologs of female reproductive genes in other organisms. We obtained genes lists from studies on female reproductive genes for the following taxa: *D. simulans*, *D. melanogaster*, *D. arizonae*, *Apis melifera* and *Anopheles gambiae*[37, 40, 42–44, 77–79]. These studies either had the goal of identifying female reproductive genes or looked at expression changes in mated females compared to virgin females. Our search yielded a list of 2,952 contigs, which were then used as queries in a BLAST search against our transcriptome without thorax sequences using the tblastx algorithm and an e-value cuttoff of 10^-5^.

### Comparison of E- and Z- reproductive genes

As a final approach to examine the bursal gland and bursa copulatrix for possible roles in post-mating sexual selection or post-mating, pre-zygotic isolation, we explored patterns of gene expression between Z and E strain females. Although differential expression alone could be viewed as evidence in support of a functional relationship and mechanism underlying dysfunctional inter-strain oviposition [59], here we emphasize enriched or depleted functional terms. After identifying differentially expressed genes between E and Z strain bursas and bursal glands respectively, we used a two-tailed fisher’s exact test (cutoff of FDR ≤ 0.05) to identify relevant GO categories and genes that might account for reduced fecundity after between-strain matings [67, 68].

## Results

### De novo sequence assembly

Single-end Illumina sequencing of 11 ECB female reproductive tissue samples yielded more than ~ 700 million raw reads. Paired-end Illumina sequencing of 2 ECB thorax samples yielded ~ 6 million raw reads. The assembled transcriptome of all 13 libraries contained 92,335 transcripts belonging to ~ 51,000 loci with a mean sequence length of 991 bp and a minimum of 201 bp (Table 1). This is likely an overestimate of the number of loci represented in our transcriptome due to de novo assembly limitations. Our mean assembled transcript lengths are greater than or equal to those reported in similar studies using the same sequencing technology [80, 81]. Prior to library normalization, log-fold change of read counts between samples differed, which can bias differential expression results. GC-content of each sample also differed prior to normalization. After normalization, gene level counts and GC-content between samples were all equal across libraries (Additional file 1: Figure S2 and Additional file 2: Figure S3).

**Table 1:** **Assembly statistics**

Total # base pairs	Number of assembled sequences	Min (bp)	Median (bp)	Mean (bp)	Max (bp)	n50 (bp)	n50 length (bp)
50868239	51307	201	535	991	15912	8297	1752

Assembled library statistics for E and Z strain bursa copulatrix, bursal gland, and thorax assembly.

### Characterizing reproductive function of the bursa copulatrix and bursal gland tissue

Our first approach to examining the reproductive function of the bursa copulatrix and bursal gland was to characterize differences in gene expression between reproductive and non-reproductive tissues. A total of 2,982 transcripts were differentially expressed between the bursa copulatrix and the thorax, representing 8% of all bursa copulatrix genes, whereas 3,316 genes were differentially expressed between the bursal gland and the thorax, representing 9% of all bursal gland genes (Additional file 3: Figure S1). For gene ontology terms enriched in the bursa copulatrix, 20% corresponded to categories related to muscle activity and organization (n = 596), while the bursal gland had 3% of genes enriched for the same categories (n = 89) (Additional file 4: Table S1). The bursal gland was also enriched for 6 gene ontology categories directly related to sexual reproduction and gamete production (Additional file 4: Table S1). Of all up-regulated differentially expressed transcripts in the bursa copulatrix compared to the thorax, 2% contained a signal peptide indicating they are secreted from the bursa copulatrix and 7% contained at least one transmembrane helix, while the bursal gland and thorax comparison yielded 16% of sequences with a signal peptide and 46% with at least 1 transmembrane helix (Table 2).

**Table 2:** **Up-regulated bursa copulatrix and bursal gland genes with secretion signal peptides or transmembrane motifs**

Tissue	Secretion signal peptide ^†^	TMHMM ^‡^
Bursa copulatrix	2%	7%
Bursal gland	16%	46%

Percent of bursa copulatrix and bursal gland genes that were up-regulated compared to the female thorax and contain either a secretion signal peptide or at least one transmembrane motif. The bursal gland has a higher percent of predicted proteins with secretion signal peptides and transmembrane helices.

^†^Number of putative proteins with secretion signal peptides.

^‡^Number of genes with at least one transmembrane helix.

Next, we examined the results from our cross-species female reproductive gene comparison to identify conserved classes of female reproductive genes across insect taxa. We found 23 putative ECB homologs in *D. simulans, D. melanogaster, D. arizonae*, and *A. melifera*, but no homologous sequences in *A.gambiae* (Table 3). Across all four species with significant blast hits to ECB bursa copulatrix or bursal gland genes, many possessed gene ontology functions related to muscle contraction (Table 3).

**Table 3:** **Between-species comparison of female reproductive genes**

Gene name	Flybase ID	ECB gene	Molecular function	Homologous gene found in
Actin 57B	FBgn0000044	comp26239_c0_seq1	Structural constituent of cytoskeleton	Drosophila simulans, Drosophila arizonae, Drosophila melanogaster
Actin 5C	FBgn0000042	comp26239_c0_seq1	Structural constituent of cytoskeleton	Drosophila simulans, Drosophila arizonae, Drosophila melanogaster
Actin 87E	FBgn0000046	comp26239_c0_seq1	Expressed in larval muscle	Drosophila simulans, Drosophila arizonae, Drosophila melanogaster
NADH dehydrogenase subunit 4	FBgn0262952	comp26239_c0_seq1, comp25774_c0_seq1	NADH dehydrogenase activity	Drosophila simulans, Drosophila arizonae, Drosophila melanogaster
Ribosomal protein LP0	FBgn0000100	comp6763_c0_seq1, comp26468_c0_seq1	Structural constituent of ribosome	Drosophila simulans
Ribosomal protein 5a	FBgn0002590	comp26375_c0_seq1, comp6724_c0_seq1	Structural constituent of ribosome	Drosophila simulans
Aldolase	FBgn0000064	comp13686_c0_seq1	Fructose-bisphosphate aldolase activity	Drosophila simulans, Drosophila melanogaster
Alpha spectrin	FBgn0250789	comp13976_c0_seq1	Actin binding	Drosophila simulans
Myosin heavy chain	FBgn0026059	comp26288_c0_seq1	Actin/ATP binding	Drosophila simulans
Alpha tubulin	FBgn0003884	comp26314_c0_seq1	GTP binding	Drosophila simulans
Calcium ATPase	FBgn0263006	comp6792_c0_seq1	Calcium-transporting ATPase activity	Drosophila simulans
Culex quinquefascitaus transport protein	FBgn0021953	comp15624_c0_seq1	Catalytic activity	Drosophila simulans
Receptor of activated protein kinase C1	FBgn0000273	comp7107_c0_seq1	ATP binding	Drosophila simulans
Elongation factor 2B	FBgn0000559	comp26371_c0_seq1	GTP binding	Drosophila simulans, Drosophila arizonae
Heat shock protein 83	FBgn0001233	comp18191_c0_seq1	ATP binding	Drosophila simulans, Drosophila melanogaster
Ribosomal protein L3	FBgn0020910	comp26384_c0_seq1	Structural constituent of ribosome	Drosophila simulans
V-ATPase	FBgn0027779	comp18376_c0_seq1	Proton-transporting ATPase activity	Drosophila melanogaster
Ribosomal protein L4	FBgn0003279	comp6770_c0_seq1	Structural constituent of ribosome	Drosophila melanogaster
Topomyosin 1	FBgn0003721	comp6703_c0_seq1	Actin binding	Drosophila melanogaster
Protein C kinase 98E	FBgn0003093	comp309823_c0_seq1	ATP binding	Drosophila melanogaster
ATPase	FBgn0013672	comp26423_c0_seq1	Hydrogen-exporting ATPase activity	Drosophila arizonae
Beta Tubulin 56D	FBgn0003887	comp13374_c0_seq1	GTP binding	Drosophila arizonae, Apis melifera
Ubiquitin	FBgn0010288	comp26322_c0_seq1	Ubiquitin thiolesterase activity	Drosophila arizonae

Comparison between ECB female reproductive genes and female reproductive genes identified in other insect taxa. The gene function category represents the top blast hit for the gene that ECB show homology to.

### Comparison of E- and Z- reproductive genes

To identify female reproductive genes that may be contributing to reproductive isolation between Z and E ECB strains, we searched for differentially expressed genes between strains. Between the Z and E strain bursa copulatrix tissues, there were 864 genes with significant differential expression and for bursal gland tissues we found 1,390 significantly differentially expressed genes between strains (Additional file 3: Figure S1).

**Figure 3:**
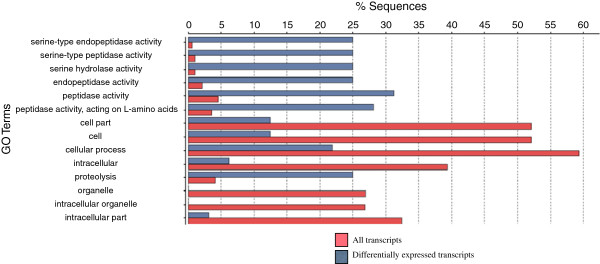
**Enriched and depleted gene ontology categories in the ECB bursa copulatrix.** Fisher’s exact test results for enriched and depleted gene ontology categories found in the bursa copulatrix compared to the entire transcriptome. All enriched gene ontology categories were related to protease activity in the bursa copulatrix except for. Pink bars represent the gene ontology categories found in the entire transcriptome, while the gray bars represent the gene ontology categories found for the genes differentially expressed between E- and Z- strains of ECB.

Subsequently, we examined enriched and depleted gene ontology classes with genes that were differentially expressed between ECB strains in either the bursa copulatrix or the bursal gland. Here, we found 7 gene ontology categories, including proteolysis and serine-type peptidase and endopeptidase activity, which were significantly enriched in the bursa copulatrix compared to our transcriptome and 7 that were depleted (Figure 3). For the bursal gland, we found one gene ontology class, structural constituent of cuticle, that was enriched compared to our transcriptome and one gene ontology category, intracellular, that was depleted.

To further explore female reproductive genes that were differentially expressed between strains, we examined our gene ontology lists for proteases and protease inhibitors. We focused on these classes because they mediate male–female post-mating interactions in Drosophila and are rapidly evolving, which makes these proteins likely to be involved in sexual conflict [82, 83]. By manually searching annotations lists after a Fisher’s exact test was run in Blast2go, we found 44 proteases and 7 protease inhibitors with differential expression in both strains and in both tissues combined (Tables 4 and 5).

**Table 4:** **Differentially expressed proteases and protease inhibitors in the bursa copulatrix**

Bursa copulatrix sequence name	Homologous protein	Direction of differential expression	Predicted function
4699	Astacin-like metalloendopeptidase	Up in E	Peptidase activity
6966	Trypsin	Up in E	Peptidase activity
333327	Prophenol oxidase activating enzyme 3	Up in E	Peptidase activity
12088	Seminal fluid protein hacp002	Up in E	Serine-type endopeptidase activity
215925	Serine protease 44-like	Up in E	Catalytic activity
100535	Trypsin zeta-like	Up in E	Catalytic activity
23804	Trypsin 7	Up in E	Hydrolase activity
10553	Cationic trypsin-like	Up in E	Hydrolase activity
5306	Seminal fluid protein hacp002	Up in E	Proteolysis
19982	Pacifastin-related serine protease inhibitor precursor	Up in E	Peptidase inhibitor activity
25107	Pacifastin-related serine protease inhibitor precursor	Up in E	Peptidase inhibitor activity
18635	Pacifastin-related serine protease inhibitor precursor	Up in E	Peptidase inhibitor activity
30890	Serine proteinase	Up in E	Peptidase activity
114622	Zinc carboxypeptidase	Up in E	Metallopeptidase activity
298440	Carboxypeptidase a5	Up in E	Peptidase activity
966	Carboxypeptidase b-like	Up in E	Metallopeptidase activity
72215	Serine protease	Up in E	Serine-type endopeptidase activity
20820	Prism serine protease inhibitor 1	Up in E	Protease inhibitor
83118	Secreted trypsin-like serine protease	Up in E	Serine-type endopeptidase activity
295616	Angiotensin converting enzyme	Up in E	Metallopeptidase activity
14604	Chymotrypsin-like protein	Up in E	Serine-type endopeptidase activity
121815	Serine protease 48	Up in E	Serine-type endopeptidase activity
18651	Tryptase 5	Up in E	Serine-type endopeptidase activity
25933	Nas-15 protein	Up in E	Metallopeptidase activity
46256	Zinc metalloproteinase nas-13 like	Up in E	Metallopeptidase activity
4699	Astacin-like metalloendopeptidase	Up in E	Peptidase activity
67637	Transmembrane protease serine 3	Up in E	Serine-type endopeptidase activity
4699	Astacin-like metalloendopeptidase	Up in E	Peptidase activity
9559	Trypsin like protein	Up in Z	Peptidase activity
31807	Neuronal pentraxin-2	Up in Z	Proteolysis
96526	Serine protease 1	Up in Z	Hydrolase activity
26516	Pancreatic trypsin inhibitor-like	Up in Z	Serine-type endopeptidase activity
12015	Colostrum trypsin	Up in Z	Serine-type endopeptidase inhibitor activity
254040	Angiotensin converting enzyme	Up in Z	Metallopeptidase activity
10123	Serine protease	Up in Z	Serine-type endopeptidase activity

ECB sequences that showed differentially expressed proteases and protease inhibitors between strains (FDR < 0.05) for the bursa copulatrix. Of 34 proteases and protease inhibitors in the bursa copulatrix, 7 were up-regulated in the Z-strain.

**Table 5:** **Differentially expressed proteases and protease inhibitors in the bursal gland**

Bursal gland sequence name	Homologous protein	Direction of differential expression	Predicted function
15139	Retinoid-inducible serine carboxypeptidase-like	Up in E	Serine-type carboxypeptidase activity
144499	Clip domain serine protease 11 precursor	Up in E	Serine-type endopeptidase activity
32437	Wap four-disulfide core domain protein 2 precursor	Up in E	Peptidase inhibitor activity
24588	Cathepsin b	Up in E	Peptidase activity
26860	Serine protease	Up in E	Serine-type endopeptidase activity
6047	Serine protease easter-like	Up in E	Peptidase activity
26327	Vitellin-degrading protease precursor	Up in E	Proteolysis
78030	Serine protease 24	Up in E	Serine-type peptidase activity
26312	Vitellin-degrading protease precursor	Up in E	Proteolysis
26502	Vitellin-degrading protease precursor	Up in E	Proteolysis
7294	Seminal fluid protein hacp057	Up in Z	Cysteine-type peptidase activity
12342	bcp inhibitor	Up in Z	Cysteine-type peptidase activity
13247	Seminal fluid protein hacp057	Up in Z	Cysteine-type peptidase activity
21908	Seminal fluid protein hacp001	Up in Z	Serine-type endopeptidase activity
29657	Trypsin inhibitor precursor	Up in Z	Peptidase inhibitor activity
31807	Neuronal pentraxin-2	Up in Z	Proteolysis
10123	Serine protease	Up in Z	Serine-type endopeptidase activity

Footnote for Table 5: ECB sequences that showed differentially expressed proteases and protease inhibitors between strains (FDR < 0.05) for the bursal gland. Of 17 proteases and protease inhibitors in the bursal gland, 7 were up-regulated in the Z-strain.

## Discussion

This study represents the first comprehensive characterization of female reproductive genes in any lepidopteran system. Using RNAseq, we identified female reproductive transcripts from the bursa copulatrix and bursal gland and from Z and E strain ECB and we characterized genes that may be involved in reproductive isolation between strains. The bursa copulatrix appears to act as a muscular sac, but it does not seem to secrete the variety of proteins found in the bursal gland, many of which are directly related to reproduction. We also found that most differentially expressed genes in the bursa copulatrix and many in the bursal gland were proteases, which could be important in post-mating sexual selection and post-mating, pre-zygotic barriers. These are of particular interest because in other species, proteases are known to be involved in female interactions with male sperm and male seminal fluid proteins [82].

### Bursa copulatrix

As the site of initial interaction between male ejaculate and the female reproductive tract [57], the bursa copulatrix is likely to be an important arena for sexual conflict. Previous work has shown that the signum helps break open the male spermatophore [36], and within Papilionidae butterflies signum complexity correlates with the thickness of the outer covering on the spermatophore [84, 85]. However, a recent study on the microstructure of the bursa copulatrix in Tortricidae moths identified pores that were suggested to perform a secretory function [56], such as processing the spermatophore.

The transcriptome of the bursa copulatrix would appear to support its role as a site for the mechanical disruption of the spermatophore. Up-regulated genes in the bursa copultrix compared to the female thorax were statistically significantly enriched for GO classes related to muscle structure or activity (Additional file 4: Table S1), and many of the female reproductive genes in flies and honey bee showing homology with ECB sequences had muscle contraction functions (Table 3). For example, bursa copulatrix transcripts comp6763_c0_seq1, comp26288_c0_seq1, and comp6703_c0_seq1 had strong hits to actin 57B, myosin heavy chain, and topomyosin 1 respectively (Table 3), all key genes during muscle contraction [86]. Similar patterns were found in the reproductive tract of female *D. melanogaster*, in which muscle contraction genes were up-regulated in response to mating [38]. Such consistent results across flies, honeybees, and moths suggest a conserved function of the female reproductive tract for muscle contraction across insect taxa. Indeed, even in taxa that lack male spermatophores, muscle contraction in the female reproductive tract has been shown to be important in moving sperm into storage and for processing male ejaculates [45].

In contrast, we found little evidence supporting a secretory role for the bursa copulatrix in ECB moths, at least for virgin females. Many of the genes that were up-regulated in the bursa copulatrix compared to the female thorax were not putatively secreted proteins (2%), consistent with the notion that the bursa copulatrix lacks an important secretory function. Nevertheless, we did find that 7% of up-regulated transcripts possessed transmembrane motifs (Table 2), suggesting the presence of receptors or membrane channels that could interact with male-derived proteins.

### Bursal gland

Prior to this study, the function of the conspicuous bursal gland present in many lepidopteran female reproductive tracts was completely unknown [49]. Given the direct connection between the bursa copulatrix and the bursal gland, we hypothesized that male-derived products could interact with the bursal gland in two possible ways: by female gland secretions moving into the bursa copulatrix, or by male ejaculate moving into the bursal gland from the bursa copulatrix. Both mechanisms are supported by our transcriptome results, with 16% of up-regulated bursal gland transcripts having secretion signal peptides and 46% having transmembrane motifs (Table 2). Furthermore, unlike the bursa copulatrix the bursal gland had fewer enriched functional categories related to muscle contraction when compared to female thorax (Additional file 4: Table S1), again suggesting mechanical spermatophore breakdown by the bursa copulatrix.

Gene expression in the bursal gland was statistically significantly enriched for many gene ontology categories related to reproduction, including sexual reproduction, gamete generation, multicellular organism reproduction, cellular process involved in reproduction, and developmental processes involved in reproduction when compared to the female thorax (Additional file 4: Table S1). Of these, one ECB female putative protein stands out. This transcript of interest showed homology to purity essence, which has been shown to be involved in sperm individualization and male fertility [87]. Although its specific role in females is unknown, finding this product in a female reproductive tissue suggests that it also plays a role in female reproduction or fertility.

### Sexual selection & reproductive isolation

Sexual conflict may extend beyond spermatophore breakdown, with this reproductive “arms race” continuing as males and females struggle for control over fertilization [88]. Such antagonistic sexual coevolution has the potential to contribute to divergence between closely related populations [8, 89]. Proteases and protease inhibitors are two classes of proteins that have been shown to be under positive selection in male reproductive tracts in *Drosophila sp*. [82, 90]. Although relatively few studies of female reproductive genes have been conducted thus far, these also suggest that proteases and protease inhibitors are important in male–female molecular interactions [42, 43, 83, 91]. Proteases found in the male ejaculate and female reproductive tract have been predicted to co-regulate expression through activation or inhibition of proteolysis, or limit the time for which a reproductive gene or protein is able to act [24, 82]. For example, in *D. arizonae* 12 digestive proteases were specifically expressed in the female reproductive tract and demonstrated signs of positive selection [92]. The functional role of these proteases is unknown; however the adaptive evolution of digestive proteases in *D. arizonae* indicates that they likely play a role in male–female molecular interactions [92].

In ECB females, 2,254 transcripts were differentially expressed between the Z and E strains of ECB in either the bursa copulatrix and the bursal gland. Within the bursa copulatrix, 86% (6/7) of statistically significantly enriched categories dealt with protease function and 34 transcripts showed significant homology to proteases or protease inhibitors (Figure 3). Seven of the proteases found in the bursa copulatrix were over-expressed in Z-strain females compared to E-strain females (Table 4), as were seven of the proteases found in the bursal gland (Table 5). In E-strain female bursa copulatrix tissues, comp18651_c0_seq1 had increased expression with a log fold change of 10 and showed significant homology to tryptase 5 (Table 4). Tryptase 5 has been shown to decrease male spermatozoa motility in humans and may be involved in fertility [93]. Another interesting protein, pacifastin-related serine protease inhibitor, was also found to be up-regulated in E-strain female bursa copulatrix tissues (Table 4). Pacifastins have been shown to regulate the immune response, reproduction and phase transition in many insects [94]. Some proteins and inhibitors in the pacifastin family have been shown to have species-specificity in locusts, suggesting they could be important in reproductive isolation [95]. Differential expression of these proteases between strains has the potential to help explain the significant reduction in egg-laying that has been documented when Z-strain females mate with E-strain males during cross-strain matings, but further research using mated females is required to make any conclusions regarding this matter. Other proteases that were differentially expressed between ECB strains in both the bursa copulatrix and the bursal gland were serine and serine-like proteases. Serine proteases have been implicated in increased egg laying after mating seen in many organisms [82]. Serine proteases have also been linked to sperm activation and immune response [82]. This is relevant because mating and sperm storage often leads to changes in regulation of immune response in the female reproductive tract [38, 43, 96], thought to protect females against male-derived pathogens. Although we have identified these proteases in virgin females, these differentially expressed serine proteases present in the ECB female reproductive tract will provide a fruitful path for future study of post-copulatory interactions and post-mating, pre-zygotic barriers.

## Conclusions

To fully understand post-mating sexual selection and post-mating, pre-zygotic isolation we must examine reproductive transcripts and proteins in taxa with diverse reproductive structures, physiologies, and mating systems. Much has been learned from *Drosophila* concerning the molecular interplay between the sexes that takes place after mating, yet little is known about how these reproductive interactions contribute to divergence. Using the European corn borer, we examined female gene expression in the first portions of the female reproductive tract that come in contact with the male ejaculate. Thus, the sequences described here provide initial insight into male and female post-mating molecular interactions in a model for speciation. Our results indicate that sexual conflict over spermatophore breakdown and male–female molecular interactions are likely to be important in Lepidoptera. We found that the main role of the bursa copulatrix is like to be as a muscular sac that mechanically processes the male spermatophore, while the bursal gland appears to serve a secretory function, producing proteins that could interact with male reproductive proteins. We also found evidence that differential expression of proteases between recently diverged strains in both tissues may contribute to post-mating, pre-zygotic reproductive isolation. Our findings represent an important first step in understanding male–female interactions and the link between sexual selection and divergence in lepidopterans. In future studies, examining changes in gene expression profiles during spermatophore processing will provide additional insight into post-mating sexual interactions.

## Availability of supporting data

The data set supporting the results of this article is available in the NCBI Transcriptome shotgun assembly project under the accession GAVD00000000 and in the NCBI bioprojects under the accession number PRJNA232037.

## Electronic supplementary material

Additional file 1: Figure S2: Stratified box-plots of the log-fold change of read count in each library. Stratified box-plots of the log-fold change of read count before (a) and after (b) normalization for each tissue library. (PDF 51 KB)

Additional file 2: Figure S3: GC content normalization. Lowess regression of (a.) non-normalized and (b) normalized GC content. (PDF 53 KB)

Additional file 3: Figure S1: MA plots for each comparison of interest. These plots shows the tagwise log-fold-change against the log-counts per million for each gene in a tissue library. Each dot on the graph represents an individual gene. The blue lines across each plot represent a 2 fold change in expression. All red points show differentially expressed genes with a FDR < 0.01 and all black dots are genes that were not significantly differentially expressed. (PDF 128 KB)

Additional file 4: Table S1: Characterizing reproductive function of the bursa copulatrix and bursal gland tissue. Gene ontology classes in the bursa copulatrix, the bursal gland, and in both tissues compared to the female thorax. Each list of gene ontology categories is in alphabetical order. (PDF 74 KB)

## Abbreviations

ECB: European corn borer
GO: Gene ontology.
