# Supplementary figures and images for: Using RNA sequencing to characterize female reproductive genes between Z and E Strains of European Corn Borer moth (Ostrinia nubilalis)
Source: BMC Genomics. 2014 Mar 12;15(1):189. doi: 10.1186/1471-2164-15-189 (PMC4007636; doi:10.1186/1471-2164-15-189)

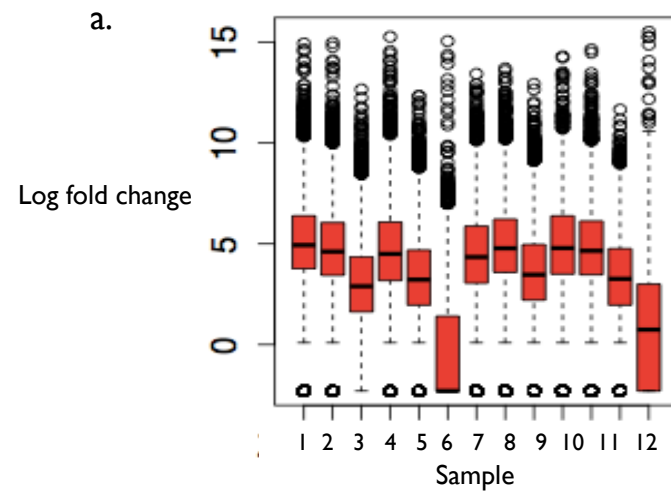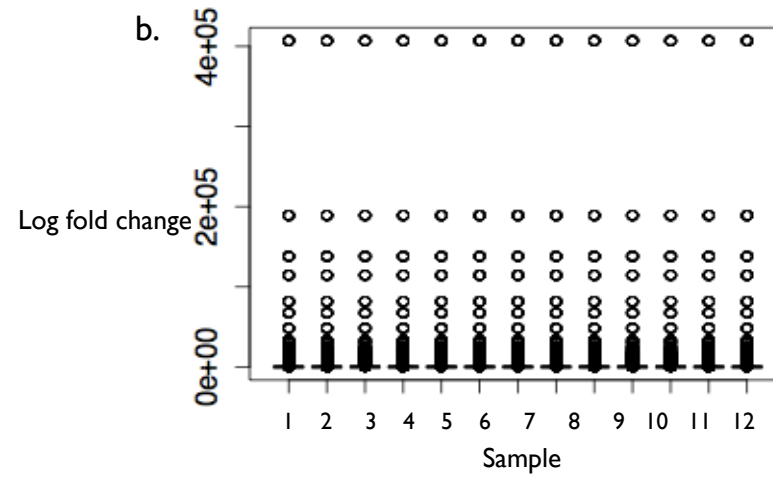

Supplement: Supplementary file 1 — Additional file 1: Figure S2: Stratified box-plots of the log-fold change of read count in each library. Stratified box-plots of the log-fold change of read count before (a) and after (b) normalization for each tissue library. (PDF 51 KB) [file 12864_2013_7027_MOESM1_ESM.pdf]

a.

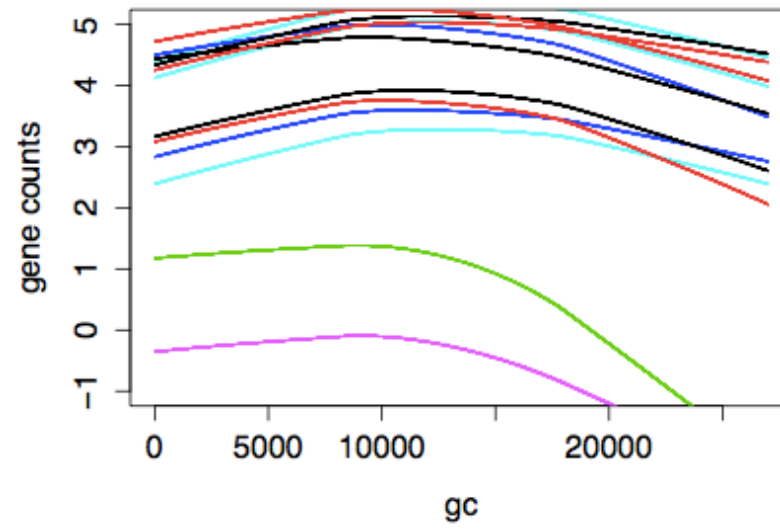

b.

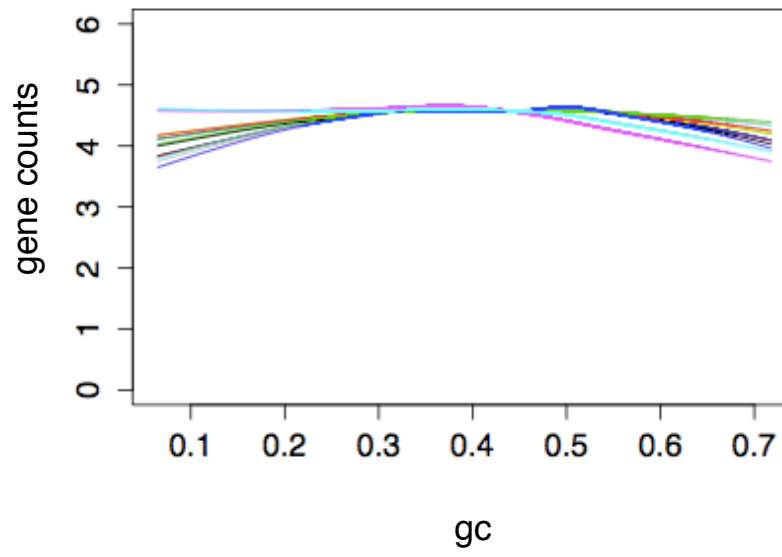

Supplement: Supplementary file 2 — Additional file 2: Figure S3: GC content normalization. Lowess regression of (a.) non-normalized and (b) normalized GC content. (PDF 53 KB) [file 12864_2013_7027_MOESM2_ESM.pdf]

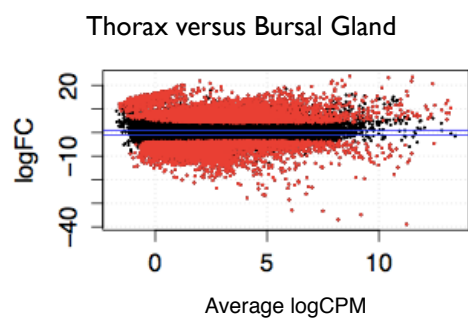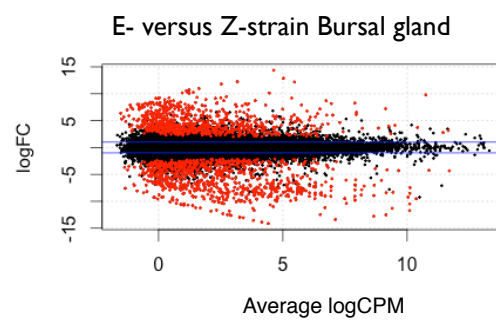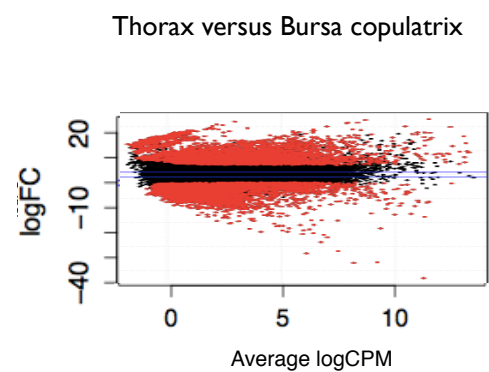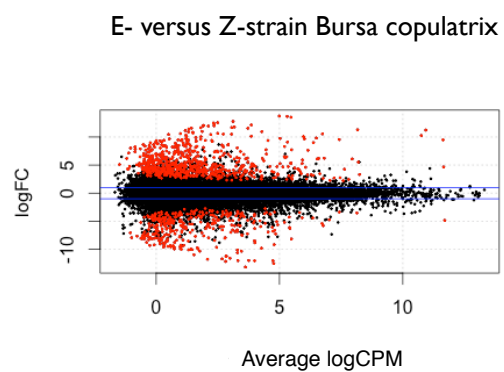

Supplement: Supplementary file 3 — Additional file 3: Figure S1: MA plots for each comparison of interest. These plots shows the tagwise log-fold-change against the log-counts per million for each gene in a tissue library. Each dot on the graph represents an individual gene. The blue lines across each plot represent a 2 fold change in expression. All red points show differentially expressed genes with a FDR < 0.01 and all black dots are genes that were not significantly differentially expressed. (PDF 128 KB) [file 12864_2013_7027_MOESM3_ESM.pdf]
