# Supplementary material for: Using RNA sequencing to characterize female reproductive genes between Z and E Strains of European Corn Borer moth (Ostrinia nubilalis)
Source: BMC Genomics. 2014 Mar 12;15(1):189. doi: 10.1186/1471-2164-15-189 (PMC4007636; doi:10.1186/1471-2164-15-189)
Supplement: Supplementary file 4 — Additional file 4: Table S1: Characterizing reproductive function of the bursa copulatrix and bursal gland tissue. Gene ontology classes in the bursa copulatrix, the bursal gland, and in both tissues compared to the female thorax. Each list of gene ontology categories is in alphabetical order. (PDF 74 KB) [file 12864_2013_7027_MOESM4_ESM.pdf]

Supplementary table 1. **Characterizing Reproductive Function of the Bursa copulatrix and Bursal gland Tissue.** Gene ontology classes in the bursa copulatrix, the bursal gland, and in both tissues compared to the female thorax. Each list of gene ontology categories is in alphabetical order.

Supplementary Table 1.

Bursal Gland GO Term	Bursal Gland GO Term Continued	Bursa copulatrix GO Term	Both GO Term
actin binding	mitochondrial inner membrane	actomyosin structure organization	actin cytoskeleton organization
actin cytoskeleton	mitochondrial membrane part	cellular component organization or biogenesis at cellular level	actin filament-based process
adherens junction	mitochondrial respiratory chain	cellular respiration	ATP synthesis coupled electron transport
anatomical structure development	mitochondrial respiratory chain complex I	contractile fiber	cellular component organization or biogenesis
anatomical structure formation involved in morphogenesis	morphogenesis of an epithelium	contractile fiber part	cellular macromolecular complex subunit organization
anatomical structure morphogenesis	morphogenesis of embryonic epithelium	DNA metabolic process	cytoplasm
anchoring junction	multicellular organism reproduction	electron transport chain	cytoplasmic part
biological regulation	multicellular organismal development	energy derivation by oxidation of organic compounds	cytoskeleton organization
cell	multicellular organismal reproductive process	head segmentation	gene expression
cell development	NADH dehydrogenase complex	I band	generation of precursor metabolites and energy

cell differentiation	negative regulation of biological process	intracellular non-membrane-bounded organelle	intracellular
cell migration	negative regulation of cellular process	mitochondrial ATP synthesis coupled electron transport	intracellular organelle
cell motility	nucleus	mitochondrial matrix	intracellular organelle part
cell part	organ development	mitochondrial part	intracellular part
cellular component organization	organ morphogenesis	mitochondrion	macromolecular complex
cellular component organization at cellular level	organelle envelope	muscle cell differentiation	NADH dehydrogenase (quinone) activity
cellular component organization or biogenesis at cellular level	organelle inner membrane	myofibril	NADH dehydrogenase (ubiquinone) activity
cellular developmental process	oxidoreductase activity, acting on NADH or NADPH	myofibril assembly	NADH dehydrogenase activity
cellular process	post-embryonic morphogenesis	non-membrane-bounded organelle	organelle
cellular process involved in reproduction	post-embryonic organ morphogenesis	respiratory electron transport chain	organelle organization
cellular protein metabolic process	protein complex	ribosome	organelle part
cytoskeletal protein binding	protein localization	sarcomere	oxidative phosphorylation
developmental process	protein modification by small protein conjugation	sarcomere organization	oxidoreductase activity, acting on NADH or NADPH, quinone or similar compound as acceptor

developmental process involved in reproduction	protein modification by small protein conjugation or removal	striated muscle cell differentiation	
dorsal closure	protein modification by small protein removal	structural constituent of ribosome	
embryonic morphogenesis	protein transporter activity	structural molecule activity	
establishment or maintenance of cell polarity	regulation of biological process	translation	
gamete generation	regulation of cellular component organization	Z disc	
germ cell development	regulation of cellular process		
Golgi apparatus	respiratory chain		
imaginal disc development	respiratory chain complex I		
imaginal disc morphogenesis	rhabdomere development		
initiation of dorsal closure	sensory organ development		
instar larval or pupal development	sexual reproduction		
instar larval or pupal morphogenesis	small GTPase mediated signal transduction		
intracellular membrane-bounded organelle	system development		
localization	tissue development		
localization of cell	tissue morphogenesis		

macromolecule localization	tube development		
membrane-bounded organelle	tube morphogenesis		
metamorphosis			
mitochondrial envelope			
